# Relaxation Effect of Abacavir on Rat Basilar Arteries

**DOI:** 10.1371/journal.pone.0123043

**Published:** 2015-04-08

**Authors:** Rachel Wai Sum Li, Cui Yang, Shun Wan Chan, Maggie Pui Man Hoi, Simon Ming Yuen Lee, Yiu Wa Kwan, George Pak Heng Leung

**Affiliations:** 1 Department of Pharmacology and Pharmacy, Faculty of Medicine, The University of Hong Kong, Hong Kong, China; 2 Key Laboratory of Ethnic Medicine Resource Chemistry (Yunnan University of Nationalities), State Ethnic Affairs Commission & Ministry of Education, School of Chemistry & Biotechnology, Yunnan University of Nationalities, Kunming, China; 3 State Key Laboratory of Chinese Medicine and Molecular Pharmacology, Department of Applied Biology and Chemical Technology, The Hong Kong Polytechnic University, Hong Kong, China; 4 Institute of Chinese Medical Sciences, University of Macau, Macao, China; 5 School of Biomedical Sciences, Faculty of Medicine, The Chinese University of Hong Kong, Hong Kong, China; Charité Universitätsmedizin Berlin, GERMANY

## Abstract

**Background:**

The use of abacavir has been linked with increased cardiovascular risk in patients with human immunodeficiency virus infection; however, the mechanism involved remains unclear. We hypothesize that abacavir may impair endothelial function. In addition, based on the structural similarity between abacavir and adenosine, we propose that abacavir may affect vascular contractility through endogenous adenosine release or adenosine receptors in blood vessels.

**Methods:**

The relaxation effect of abacavir on rat basilar arteries was studied using the myograph technique. Cyclic GMP and AMP levels were measured by immunoassay. The effects of abacavir on nucleoside transporters were studied using radiolabeled nucleoside uptake experiments. Ecto-5′ nucleotidase activity was determined by measuring the generation of inorganic phosphate using adenosine monophosphate as the substrate.

**Results:**

Abacavir induced the relaxation of rat basilar arteries in a concentration-dependent manner. This relaxation was abolished when endothelium was removed. In addition, the relaxation was diminished by the nitric oxide synthase inhibitor, L-NAME, the guanylyl cyclase inhibitor, ODQ, and the protein kinase G inhibitor, KT5820. Abacavir also increased the cGMP level in rat basilar arteries. Abacavir-induced relaxation was also abolished by adenosine A2 receptor blockers. However, abacavir had no effect on ecto-5’ nucleotidase and nucleoside transporters. Short-term and long-term treatment of abacavir did not affect acetylcholine-induced relaxation in rat basilar arteries.

**Conclusion:**

Abacavir induces acute endothelium-dependent relaxation of rat basilar arteries, probably through the activation of adenosine A2 receptors in endothelial cells, which subsequently leads to the release of nitric oxide, resulting in activation of the cyclic guanosine monophosphate/protein kinase G-dependent pathway in vascular smooth muscle cells. It is speculated that abacavir-induced cardiovascular risk may not be related to endothelial dysfunction as abacavir does not impair relaxation of blood vessels. The most likely explanation of increased cardiovascular risk may be increased platelet aggregation as suggested by other studies.

## Introduction

Use of the nucleoside reverse transcriptase inhibitor (NRTI), abacavir, in the treatment of patients infected with the human immunodeficiency virus (HIV) may be associated with increased cardiovascular risk [1]. An observational study conducted by the Data Collection on Adverse events of Anti-HIV Drugs (D:A:D) study group reported that patients exposed to abacavir had a higher risk of myocardial infarction and stroke even after adjustment for conventional cardiovascular risk factors [2]. A strong correlation between abacavir and increased risk of myocardial infarction has also been reported in other cohort studies [3–5]. However, clinical trials sponsored by the manufacturer of abacavir (GlaxoSmithKline) and other studies claim that abacavir therapy does not increase the risk of myocardial ischemic events [6–8].

The mechanism of abacavir-induced increased cardiovascular risk is unknown. Theoretically, the association between abacavir and increased cardiovascular risk may be related to endothelial dysfunction. An *in vitro* experiment demonstrated that abacavir decreases the expression of endothelial nitric oxide synthase (eNOS) and increases oxidative stress in porcine and human pulmonary arterial endothelial cells [9]. In addition, abacavir may interfere with purine signaling pathways and may lead to activation of T lymphocytes, which causes vascular damage [10]. It has been postulated that abacavir may inhibit the activity of ecto-5 nucleotidase, resulting in decreased release of the anti-inflammatory nucleoside, adenosine, and increased release of the pro-inflammatory agents, adenosine triphosphate and adenosine diphosphate [11]. Levels of inflammatory markers such as C-reactive protein, *metalloproteinase-9* and myeloperoxidase were also found to be elevated after switching antiretroviral therapy to abacavir [12]. However, several studies have reported that levels of inflammatory or coagulopathic biomarkers are not significantly increased in patients on abacavir therapy [13–16]. Abacavir, didanosine and tenofovir have no effect on coronary endothelial cell gene transcription and protein expression of proinflammatory molecules such as vascular cell adhesion molecule-1, intracellular adhesion molecule-1, monocyte chemotactic protein-1 and interleukin-6. In addition, these agents do not affect gene expression of the intracellular reactive oxygen species producing enzyme, NADPH oxidase, and the apoptosis regulating molecules [17]. Similar conflicting results were also observed in a single study in which the levels of vascular cell adhesion molecule-1 were decreased, while those of interleukin-6 were elevated during abacavir treatment [18]. Therefore, the effect of abacavir on endothelial function remains obscure. We hypothesize that abacavir may impair endothelial function. In addition, abacavir is a guanosine analog with a chemical structure similar to the physiological vasodilator adenosine. Therefore, we postulate that abacavir may affect vascular contractility by altering endogenous adenosine release or adenosine receptors in blood vessels.

## Materials and Methods

### Ethical approval of the study protocol

All experiments performed in this study were approved by the Committee on the Use of Live Animals in Teaching and Research of the University of Hong Kong.

### Animal studies

Abacavir can penetrate deep into the central nervous system [19,20] **and** remains at a detectable concentration in the brain for a considerable time [20,21]. One cohort study showed that abacavir was associated with an increased risk of cerebrovascular events [22]. Therefore, rat basilar arteries were used as the primary model of blood vessels in the present study. Male Sprague–Dawley rats (8–10 weeks; 300–350 g) were used. The rats were kept in a temperature and humidity controlled room, with free access to food and water, and maintained under a 12:*12 h light*-dark cycle. The rats were anesthetized by intraperitoneal injection of sodium pentobarbitone (70 mg/kg) with heparin (0.5 U/kg) to avoid the formation of blood clots. The brains were quickly dissected and transferred to ice-cold Krebs–Henseleit solution (KHS; composition in mM: NaCl 120, KCl 4.76, NaHCO_3_ 25, NaH_2_PO_4_ 1.18, CaCl_2_ 1.25, MgSO_4_ 1.18, glucose 5.5, at pH 7.4). Basilar arteries were isolated under a dissecting microscope. To study the short-term effect of abacavir, the isolated rat basilar arteries were incubated in culture medium containing abacavir (10 μM) at 37°C in 5% CO_2_ / 95% air for 24 h. The arteries were washed before mounting in the myograph system. To study the long-term effect of abacavir, rats were fed abacavir (16 mg/kg/day or 160 mg/kg/day, intragastrically) by gavage for 4 weeks. The 4- week period was chosen based on a previous study which showed that antiretroviral agents such as azidothymidine could induce endothelial dysfunction in rats after treatment for 30 days [23]. Abacavir was dissolved in 5% methylcellulose. Rats fed with the same volume of 5% methylcellulose served as the control group. Each group contained eight rats.

### Measurement of vascular contractility

Rings of basilar arteries were held between two 40-μm tungsten wires mounted on the jaw of a Mulvany–Halpern myograph filled with 8 mL KHS to record isometric tension. The bath solution was continuously gassed with 95% O_2_/5% CO_2_ and maintained at 37°C. Basilar artery rings were equilibrated for 1 h under 0.5 g before experimentation. The bath solution was changed every 15 min. Basal tension was readjusted if necessary.

After equilibration, the arterial rings were stimulated twice with 60 mM KCl to obtain maximal contraction. The rings were contracted using endothelin-1 (1–10 nM) to obtain maximal contraction and then relaxed with acetylcholine (10 μM). Arteries which were relaxed by more than 80% reflected the presence of endothelium. When necessary, endothelium-denuded vessels were prepared by gently rubbing the intimal surface of the rings with hair. Loss of the relaxation response to acetylcholine indicated the absence of endothelia. Drugs were removed by repeatedly changing the bath solution. After the re-establishment of baseline tension, rings were again contracted using endothelin-1 (1–10 nM), which was approximately 90% of the maximal contraction induced by 60 mM KCl. To study the mechanism of action, arterial rings were incubated with different pharmacological inhibitors for 30 min before contraction with endothelin-1. The level of contraction induced by endothelin-1 in rat basilar arteries was not significantly altered by the pharmacological agents used in the present study. Cumulative relaxation–response curves were constructed by stepwise addition of different relaxation agents at half-log basis. After the experiments, the arteries were sectioned and stained using hematoxylin and eosin to ensure that the arteries were grossly normal after the drug treatments.

### Cell culture

Human umbilical vein endothelial cells (HUVECs) were purchased from the American Type Culture Collection (Manassas, VA, USA). They were cultured in Ham's Kaighn's Modification F12K medium supplemented with 10% (v/v) fetal bovine serum (FBS), 10,000 units/mL penicillin, 10,000 μg/mL streptomycin, 25 μg/mL amphotericin, 0.1 mg/mL heparin and 0.05 mg/mL endothelial cell growth supplement.

PK15NTD/ENT1 and PK15NTD/ENT2 cells were used to study the effects of abacavir on nucleoside transporters. These cells are derived from the nucleoside transporter-deficient cell line PK15NTD and stably transfected with clone human ENT1 and ENT2 [24]. Cells were cultured in Eagle’s minimal essential medium/Earle’s balanced salt solution (1:1) with 0.1 mM non-essential amino acids, 1 mM pyruvate, 10% (v/v) FBS, 100 units/mL penicillin, 100 μg/mL streptomycin and 0.25 μg/mL amphotericin. Cells were incubated in 75 cm^2^ culture flasks at 37°C with 5% CO2/95% air. The medium was changed every 3–4 days. Confluent cells were used for further experiments.

### Uptake assay

The uptake assay was carried out as previously described [25]. Cells were washed twice with HEPES-buffered Ringer’s solution (135 mM NaCl, 5 mM KCl, 3.33 mM NaH_2_PO_4_, 0.83 mM Na_2_HPO_4_, 1.0 mM CaCl_2_, 1.0 mM MgCl_2_, 10 mM glucose, and 5 mM HEPES at pH 7.4). To study abacavir uptake in HUVECs, 300 μL of [^3^H]abacavir (10 μM, 2 μCi/mL) in HEPES-buffered solution was added to the cells at different time points at room temperature to allow [^3^H]abacavir uptake. To study the effects of abacavir on nucleoside transporters in HUVECs, ENT1/PK15NTD cells and ENT2/PK15NTD cells, [^3^H]adenosine (10 μM, 2 μCi/mL) was added to the cells for 4 min at room temperature. Different concentrations of abacavir were added simultaneously with [^3^H]adenosine. To determine the passive uptake of adenosine, monolayers of cells were incubated in buffer containing [^3^H]adenosine in the presence of 0.5 mM NBMPR (which blocks all adenosine uptake by ENT1 and ENT2). The uptake process was terminated by washing the plates rapidly with ice-cold phosphate-buffered saline (PBS) buffer. The plates were air-dried and 500 μL of 5% Triton-X was added to lyse cells overnight. Cell lysates were mixed with 2 mL of scintillation liquid. Radioactivity was measured using a β-scintillation counter.

### Determination of levels of cyclic GMP and AMP

Rat basilar arteries were incubated in oxygenated KHS at 37°C in the absence or presence of abacavir (30 μM) or ODQ (10 μM; a guanylyl cyclase inhibitor) for 20 min. cGMP and cAMP from arteries were extracted and their concentrations were determined using commercially available kits (Cyclic GMP/AMP EIA kit; Cayman Chemical, Ann Arbor, MI, USA).

### Determination of ecto-5′ nucleotidase activity

Ecto-5′ nucleotidase catalyzes the metabolism of adenosine monophosphate to adenosine to form inorganic phosphate. Therefore, the amount of inorganic phosphate formed can reflect the activity of ecto-5′ nucleotidase. The principle of this assay is based upon the measurement of inorganic phosphate, which reacts with ammonium molybdate to form a colored product at an absorbance of 750 nm. The assay procedure has been previously described [26].

### Materials

Ham's Kaighn's Modification F12K medium and Eagle’s minimal essential medium/Earle’s balanced salt were purchased from Invitrogen Life Technologies (Carlsbad, CA, USA). KT 5720 and KT 5823 were purchased from Calbiochem EMD Chemical Group (Gibbstown, NJ, USA). Abacavir and [^3^H]abacavir were purchased from Moravek Biochemicals and Radiochemicals **(Brea, CA, USA).** cGMP and cAMP EIA Kits were purchased from Cayman Chemical. AMP, indomethacin (INDO), L-NAME, ODQ, SQ 22536, TRAM-34 and UCL 1684 were purchased from Sigma–Aldrich (St. Louis, MO, USA).

### Statistics

The Kolmogorov-Smirnov test was used to examine the normal distribution of the data. Data are mean ± standard error mean (S.E.M.). The Student’s *t-*test and analysis of variance (one-way ANOVA) were used for paired and multiple variants, respectively. Concentration–response curves were fitted by a four-parameter logistic function curve, and half-maximal inhibitory concentration (IC_50_) values were determined. *P*<0.05 was considered significant. All statistical tests were undertaken using GraphPad Prism version 5.01 (GraphPad, San Diego, CA, USA).

## Results

### Endothelium-dependent relaxation induced by abacavir

**Fig 1:**
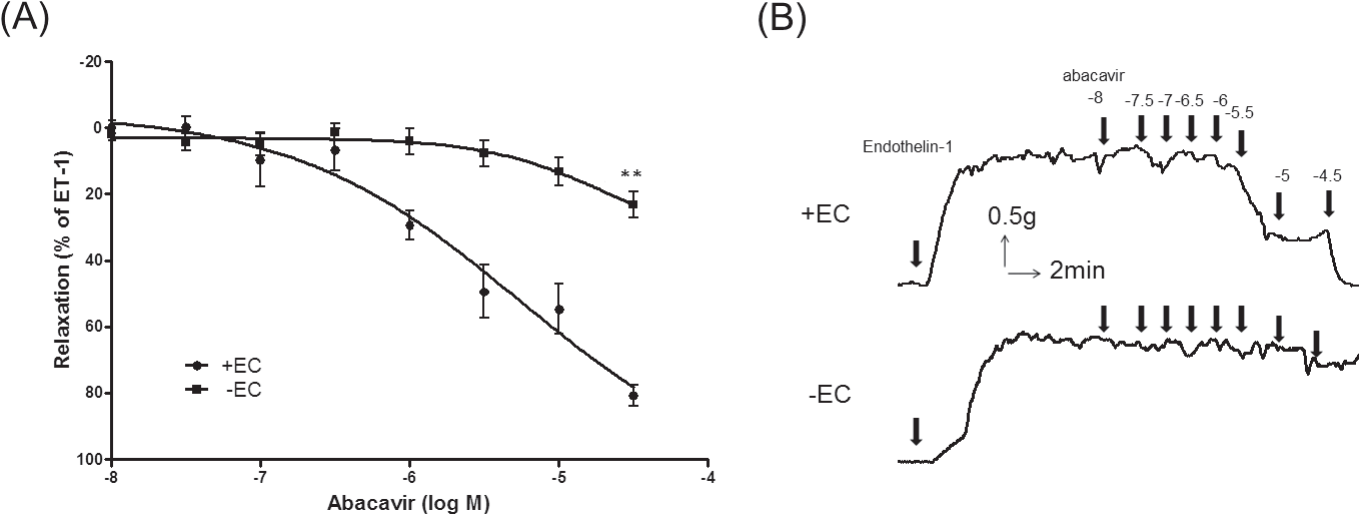
Effect of abacavir on the relaxation of rat basilar arteries. Abacavir-induced relaxation of rat basilar arteries was measured in the presence (+EC) or absence (–EC) of endothelial cells. (A) Changes in tension were expressed as the percentage decrease in response to contraction caused by endothelin-1 (ET-1; 1–10 nM). (B) Sample trace showing the effect of endothelium removal on abacavir-induced relaxation. Values are means ± S.E.M of six to eight sets of experiments and statistical comparisons were made using ANOVA. ** *P*<0.01 compared with control.

**Fig 2:**
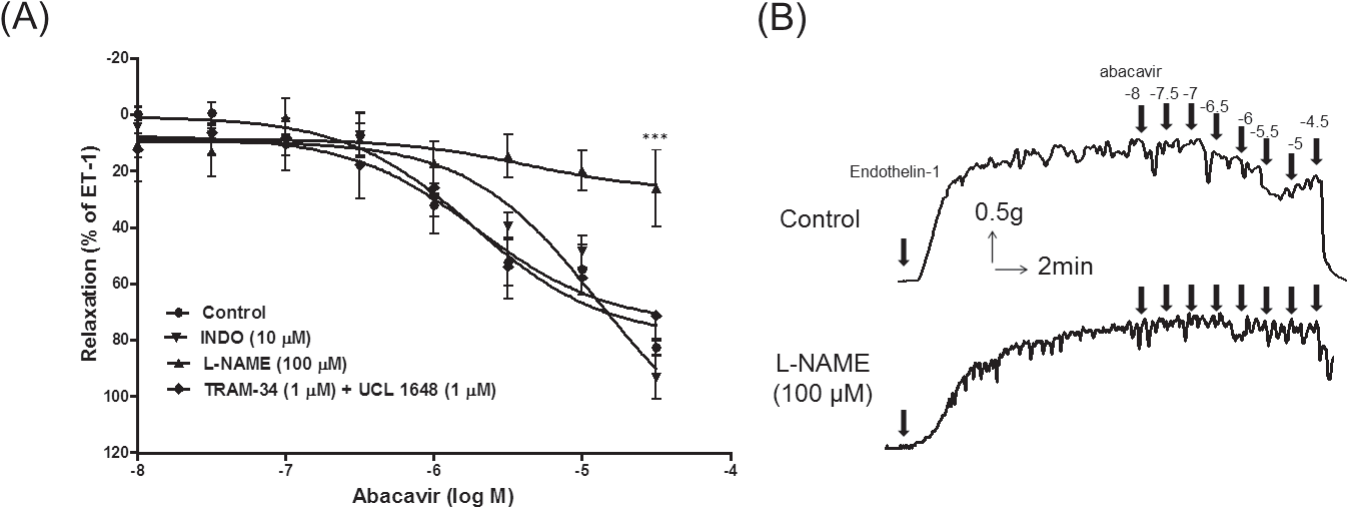
Involvement of NO, prostacyclin and EDHFs on abacavir-induced relaxation of rat basilar arteries. Rings of rat basilar arteries were treated with L-NAME (100 μM), indomethacin (INDO; 10 μM), or TRAM-34 (1 μM) plus UCL 1648 (1 μM) for 30 min. Rings without inhibitor treatment served as controls. Abacavir-induced relaxation of basilar arteries was then measured. (A) Changes in tension were expressed as the percentage decrease in response to contraction caused by endothelin-1 (ET-1; 1–10 nM). (B) Sample trace showing the effect of L-NAME on abacavir-induced relaxation. Values are means ± S.E.M of five sets of experiments and statistical comparisons were made using ANOVA. *** *P*<0.005 compared with control.

Abacavir had no contractile effect on rat basilar arteries with basal tone (data not shown). However, abacavir induced the relaxation of rat basilar arteries in a concentration-dependent manner with an IC_50_ value of 5.46 μM (Fig 1). This abacavir-induced relaxation was greatly diminished when the endothelium was removed from rat basilar arteries. It is well known that the major relaxing factors released from endothelial cells are nitric oxide (NO), prostacyclin (PGI_2_) and endothelium-derived hyperpolarizing factor (EDHF). To study which factors were responsible for the abacavir-induced relaxation response, the effects of L-NAME (100 μM; an endothelial NO synthase inhibitor), indomethacin (10 μM; a cyclooxygenase inhibitor) and TRAM-34 plus UCL 1684 (both at 1 μM; inhibitors of EDHF-mediated responses) were studied. Abacavir-induced relaxation of rat basilar arteries was not affected when arteries were pretreated with indomethacin or TRAM-34 plus UCL 1684 (Fig 2). In contrast, abacavir-induced relaxation was abolished by L-NAME.

#### Involvement of the cGMP/PKG pathway in abacavir-induced relaxation

**Fig 3:**
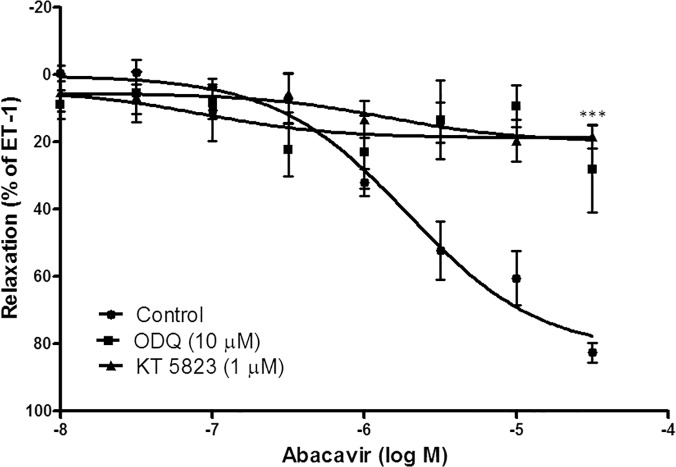
Role of the cGMP signaling pathway in abacavir-induced relaxation of rat basilar arteries. Rings of rat basilar arteries were treated with ODQ (10 μM) or KT 5823 (1 μM) for 30 min. Rings without inhibitor treatment served as controls. Abacavir-induced relaxation of basilar arteries was then measured. Changes in tension were expressed as the percentage decrease in response to contraction caused by endothelin-1 (ET-1; 1–10 nM). Values are means ± S.E.M of five sets of experiments and statistical comparisons were made using ANOVA. *** *P*<0.005 compared with control.

**Fig 4:**
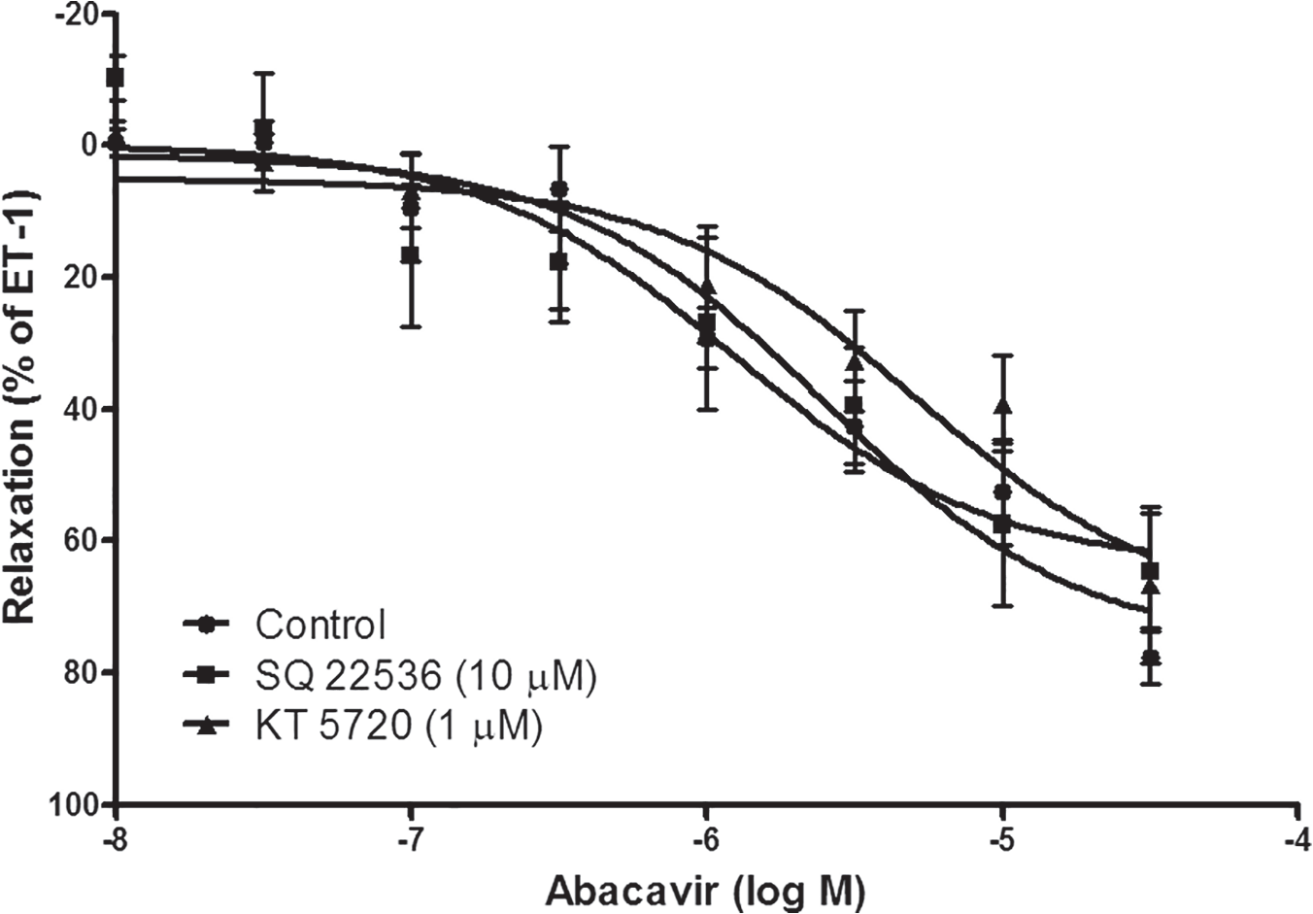
Role of the cAMP signaling pathway in abacavir-induced relaxation of rat basilar arteries. Rings of rat basilar arteries were treated with SQ 22536 (10 μM) or KT 5720 (1 μM) for 30 min. Rings without inhibitor treatment served as controls. Abacavir-induced relaxation of basilar arteries was then measured. Changes in tension were expressed as the percentage decrease in response to contraction caused by endothelin-1 (ET-1; 1–10 nM). Values are means ± S.E.M of five sets of experiments and statistical comparisons were made using ANOVA.

Abacavir-induced relaxation was examined in the presence of ODQ (10 μM; a guanylyl cyclase inhibitor) and KT 5823 (1 μM; a protein kinase G inhibitor) to study the contribution of the cGMP/PKG-dependent pathway in this process. ODQ and KT 5823 greatly reduced abacavir-induced relaxation of basilar arteries (Fig 3). In contrast, SQ 22536 (10 μM; an adenylyl cyclase inhibitor) and KT 5720 (1 μM; a protein kinase A inhibitor) had no effect on abacavir-induced relaxation of basilar arteries (Fig 4).

**Fig 5:**
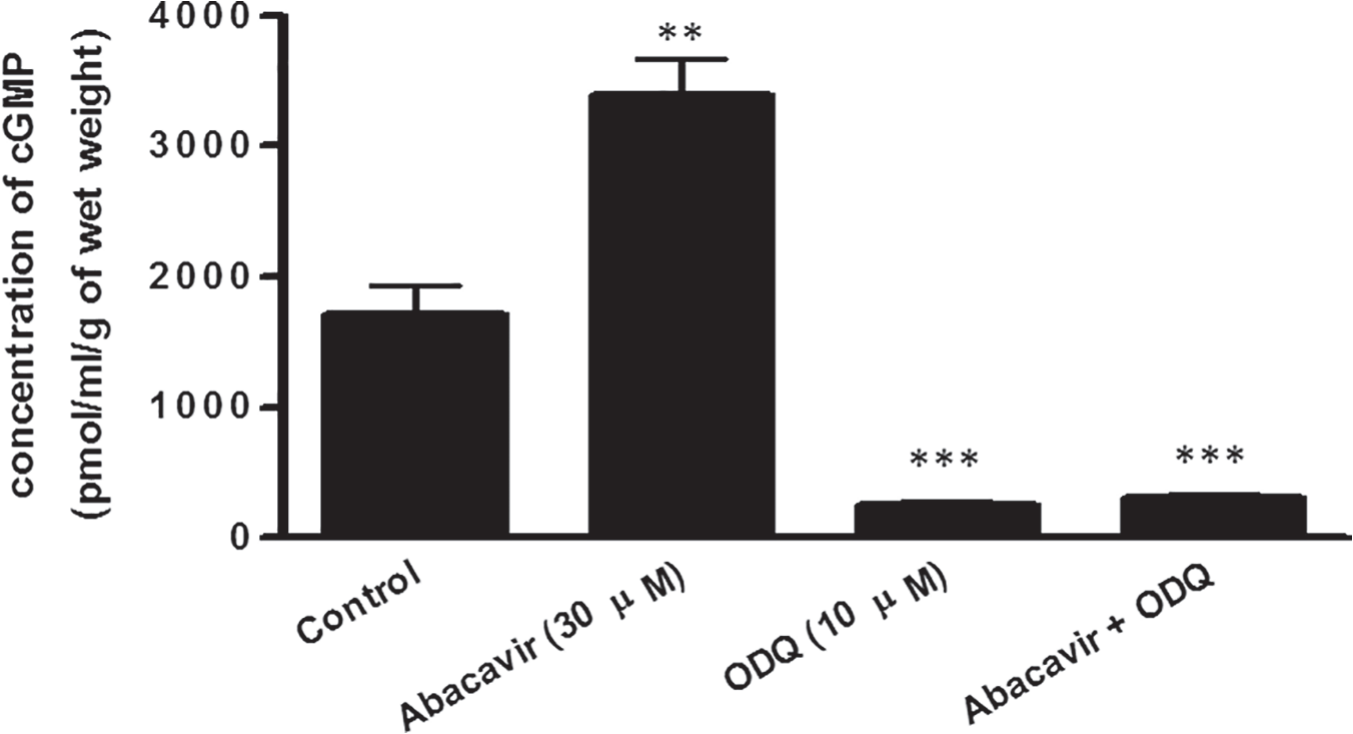
Effect of abacavir on the concentration of cGMP in rat basilar arteries. Rat basilar arteries were pretreated with or without ODQ (10 μM) for 30 min and then stimulated with or without abacavir (30 μM) for 20 min. Arteries not treated with ODQ and abacavir served as controls. cGMP content in the arteries was determined using an ELISA kit. Values are means ± S.E.M. of three sets of experiments and statistical comparisons were made using ANOVA. ** *P*<0.01 and *** *P*<0.005 compared with control.

**Fig 6:**
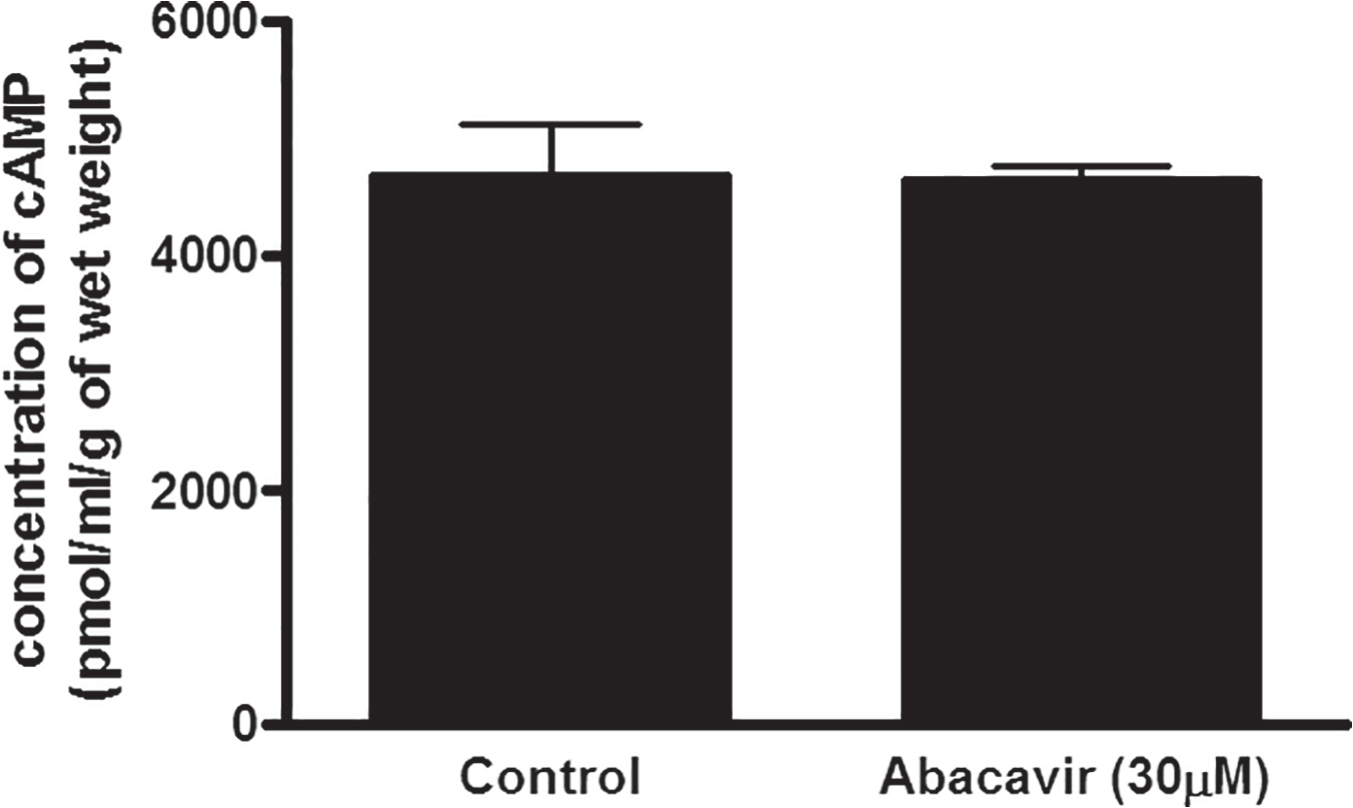
Effect of abacavir on the concentration of cAMP in rat basilar arteries. Rat basilar arteries were incubated without (control) or with 30 μM abacavir for 20 min. cAMP content in arteries was determined using an ELISA kit. Values are means ± S.E.M. of three sets of experiments and statistical comparisons were made using student’s *t*-test.

To further confirm that abacavir-induced relaxation was mediated by the cGMP signaling pathway, but not by the cAMP signaling pathway, the effects of abacavir on the levels of cGMP and cAMP in rat basilar arteries were investigated. The intracellular concentration of cGMP in rat basilar arteries was increased by abacavir (30 μM) (Fig 5). The increased level of cGMP by abacavir was diminished by ODQ (10 μM). In contrast, abacavir had no effect on cAMP level in rat basilar arteries (Fig 6).

#### Role of adenosine receptors in abacavir-induced relaxation

**Fig 7:**
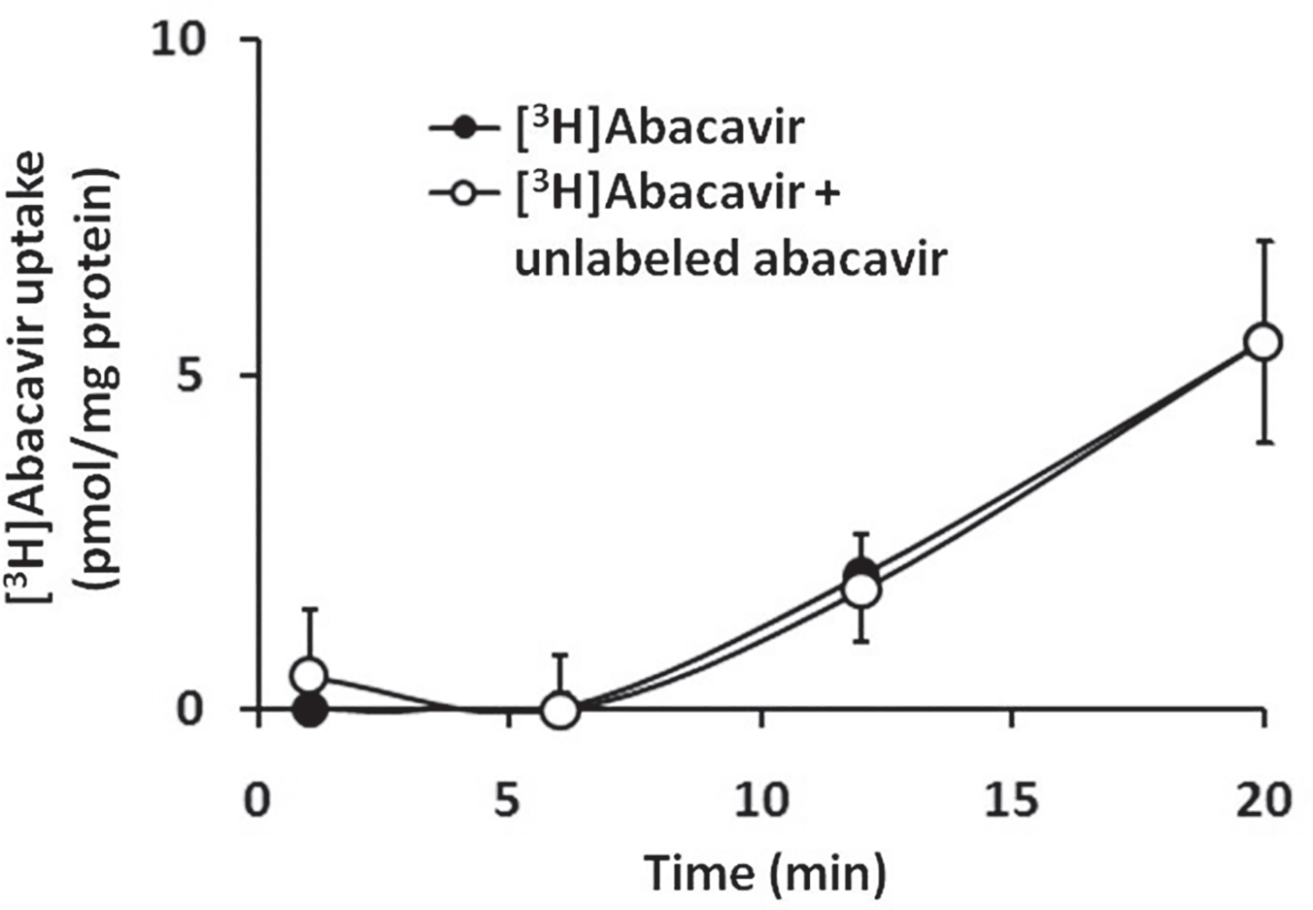
Uptake of [^3^H]abacavir by HUVECs. Uptake of [^3^H]abacavir (2 μCi/mL, 1 nM) by HUVECs was measured after incubation with [^3^H]abacavir with or without unlabeled abacavir (20 μM) for 1, 6, 12, and 20 min at room temperature. Values are means ± S.E.M. of three sets of experiments carried out in triplicate and statistical comparisons were made using ANOVA.

It is hypothesized that abacavir may be transported into endothelial cells, thereby stimulating the synthesis of NO in the latter, which induces relaxation of vascular smooth muscle cells. Abacavir uptake by HUVECs increased with time (Fig 7). However, [^3^H]abacavir uptake by HUVECs was negligible in the first 5 min. This abacavir uptake was not inhibited by an excess of non-radioactive abacavir, indicating that abacavir uptake in HUVECs was primarily mediated by passive diffusion.

**Fig 8:**
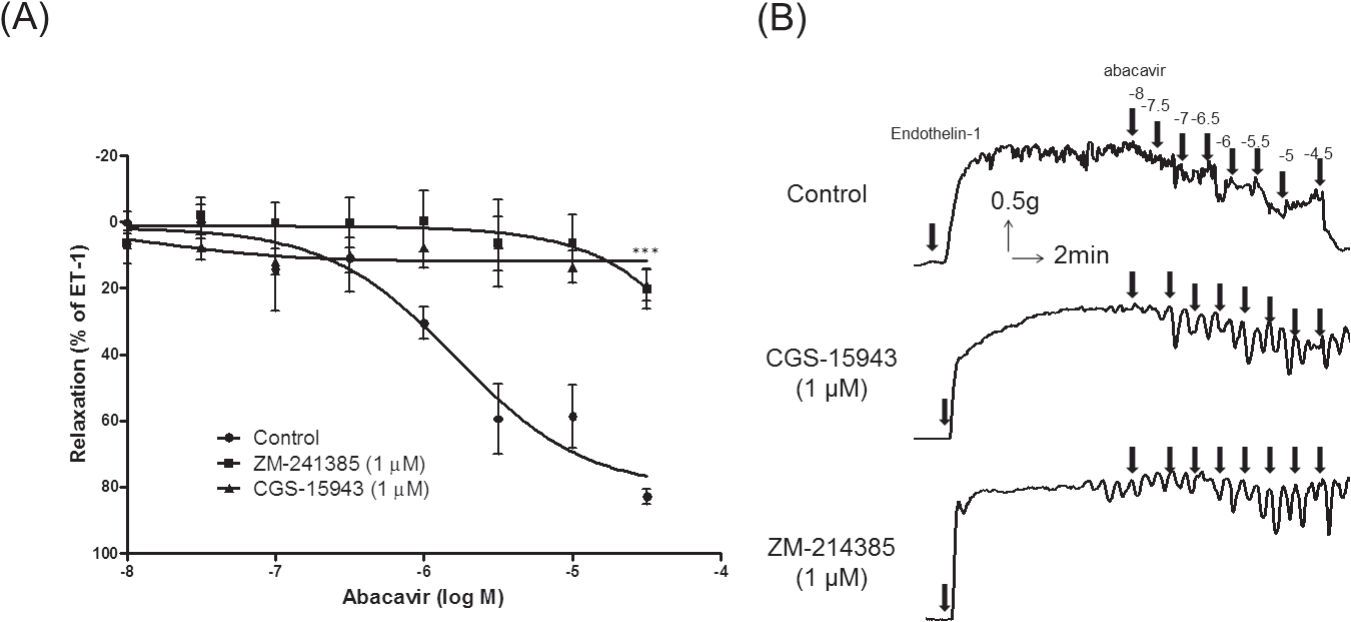
Effects of adenosine receptor antagonists on abacavir-induced relaxation of rat basilar arteries. Rings of rat basilar arteries were treated with CGS-15943 (1 μM) or ZM-241385 (1 μM) for 30 min. Rings without antagonist treatment served as controls. Abacavir-induced relaxation of rat basilar arteries was then measured. (A) Changes in tension were expressed as a percentage decrease in response to contraction caused by endothelin-1 (ET-1; 1–10 nM). (B) Sample trace showing the effects of CGS-15943 and ZM-241385 on abacavir-induced relaxation. Values are means ± S.E.M of four to five sets of experiments and statistical comparisons were made using ANOVA. ***P <0.005 versus control.

Another hypothesis is that abacavir may act on adenosine receptors to induce relaxation as abacavir is a nucleoside analog and its chemical structure is similar to that of the physiological vasodilator, adenosine [27,28]. To determine whether abacavir-induced relaxation of basilar arteries was adenosine receptor-mediated, the effects of CGS-15943 (1 μM; a non-selective adenosine receptor antagonist) and ZM-241385 (1 μM; a selective A_2_ receptor antagonist) were examined. CGS-15943 and ZM-241385 abolished the relaxation effect of abacavir (Fig 8).

**Fig 9:**
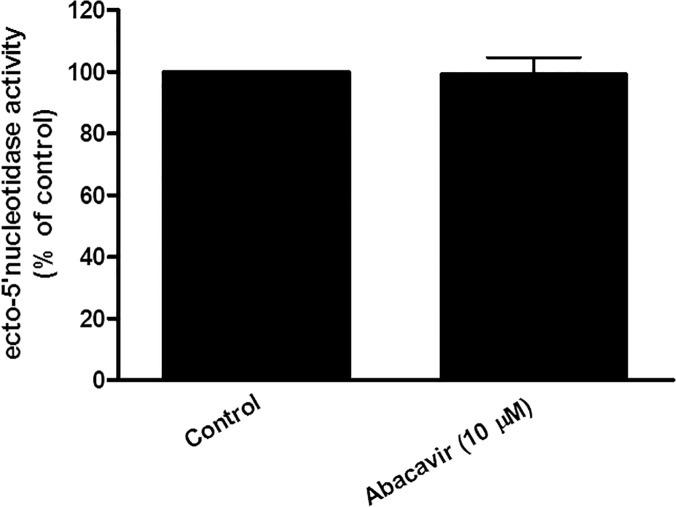
Ecto-5′-nucleotidase activity in HUVECs. HUVECs were treated without (control) or with abacavir (10 μM) for 24 h and the activity of ecto-5’ nucleotidase measured. Values are means ± S.E.M. of three sets of experiments carried out in triplicate and statistical comparisons were made using Student’s *t*-test.

**Fig 10:**
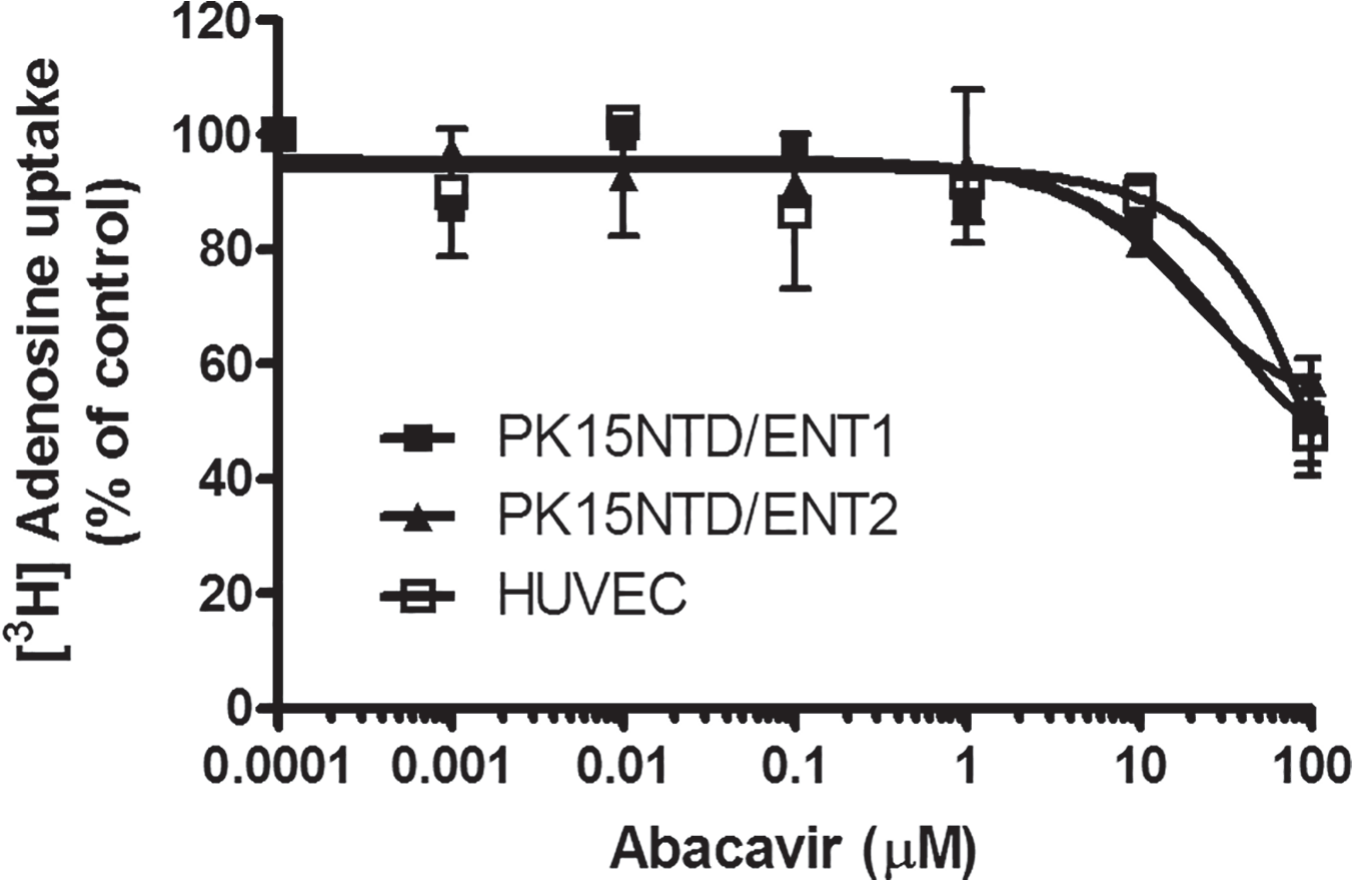
Effects of abacavir on adenosine uptake in HUVECs, PK15NTD/ENT1 cells and PK15NTD/ENT2 cells. [^3^H]adenosine uptake (10 μM, 2 μCi/mL) by HUVECs (□), PK15NTD/ENT1 cells (■) and PK15NTD/ENT2 cells (▲) was measured at room temperature for 4 min in the presence of abacavir (1 nM to 100 μM). Values are means ± S.E.M. of three experiments carried out in triplicate and statistical comparisons were made using ANOVA.

Further experiments were carried out to ascertain if abacavir could act on adenosine receptors indirectly through the simulation of ecto-5′ nucleotidase, which increased the generation of endogenous adenosine. Our results showed that the activity of ecto-5′ nucleotidase was unchanged after HUVECs were incubated with abacavir for 24 h (Fig 9). Another experiment was undertaken to determine if abacavir could inhibit nucleoside transporters, thereby reducing the disappearance of extracellular adenosine. Although adenosine uptake by HUVECs, ENT1/PK15NTD cells and ENT2/PK15NTD cells was inhibited by 100 μM abacavir by approximately 40%, abacavir had no effect on adenosine uptake when the concentration was less than 10 μM (Fig 10).

### Effect of short-term and long-term treatment with abacavir on relaxation

**Fig 11:**
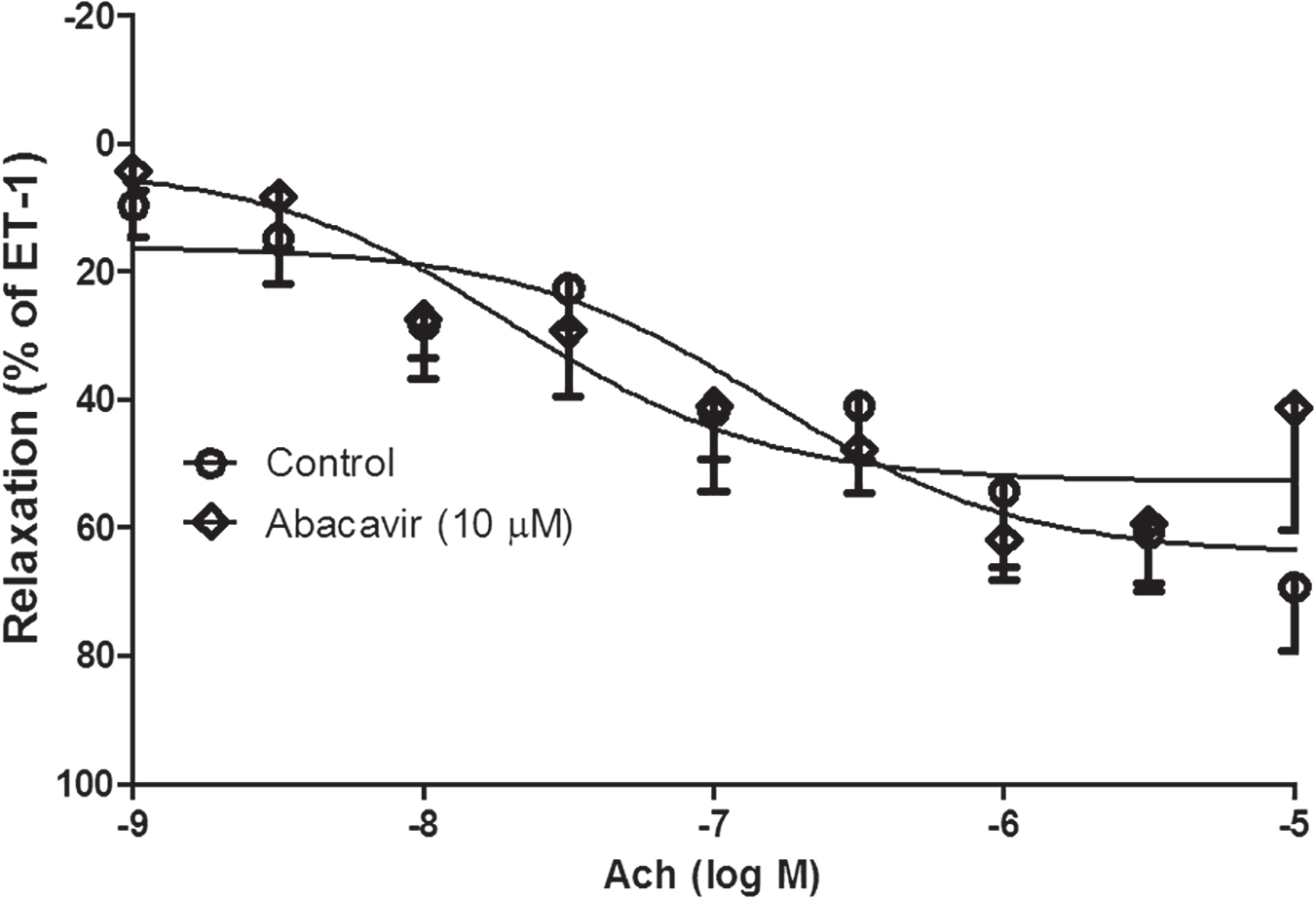
Effects of short-term treatment with abacavir on acetylcholine-induced relaxation of rat basilar arteries. Basilar arterial rings were treated with abacavir (10 μM) for 24 h. Rings not treated with abacavir served as controls. Acetylcholine (Ach)-induced relaxation of basilar arteries was then measured. Changes in tension were expressed as a percentage decrease in response to contraction caused by endothelin-1 (ET-1 1–10 nM). Values are means ± S.E.M. of five to eight sets of experiments and statistical comparisons were made using ANOVA.

**Fig 12:**
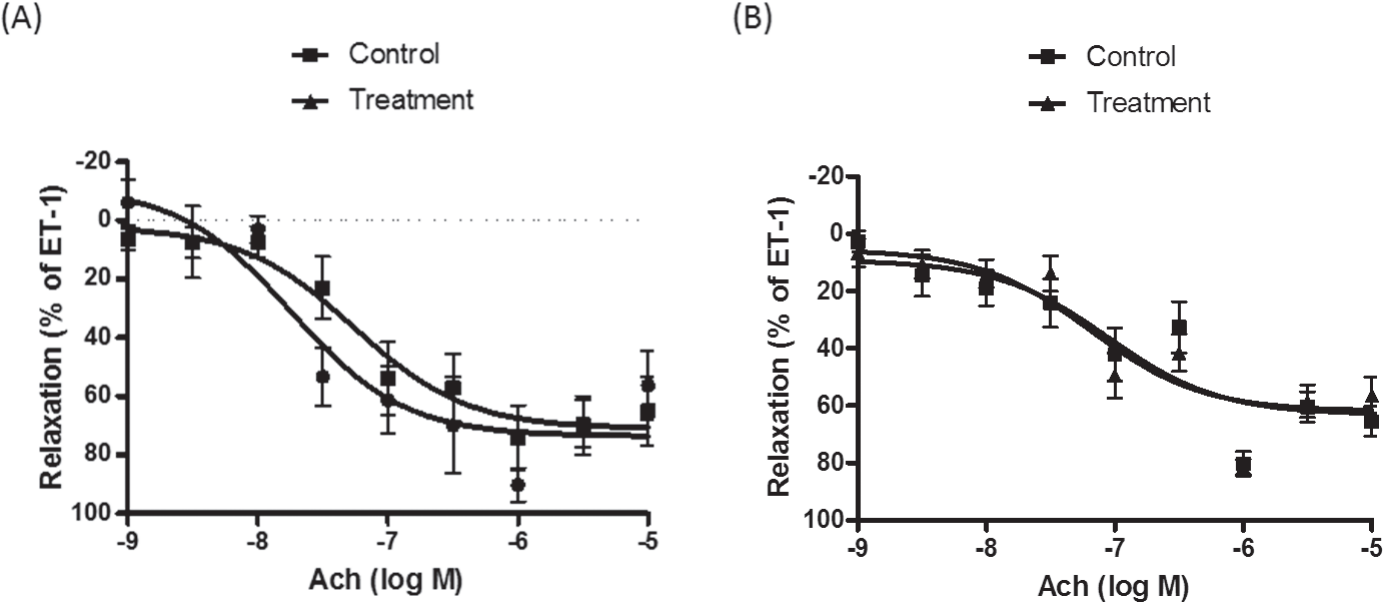
Effect of long-term treatment of abacavir on acetylcholine-induced relaxation of rat basilar arteries. Rats were treated with (A) 16 mg/kg/day of abacavir and (B) 160 mg/kg/day of abacavir for 4 weeks. Rats not treated with abacavir served as controls. Acetylcholine (Ach)-induced relaxation of basilar arteries was then measured. Changes in tension were expressed as a percentage decrease in response to contraction caused by endothelin-1 (ET-1; 1–10 nM). Values are means ± S.E.M. of eight sets of experiments and statistical comparisons were made using ANOVA.

Acetylcholine-induced endothelium-dependent relaxation was not altered when basilar arteries were pre-incubated with abacavir for 24 h (10 μM) (Fig 11). For long-term treatment, rats were fed abacavir (16 mg/kg/day or 160 mg/kg/day) for 4 weeks. The weight of the rats was 537.4 ± 12.5 g (control) vs 488.6 ± 5.9 g (treated with abacavir 16 mg/kg); and 484.3 ± 5.064 g (control) vs 479.5 ± 15.02 g (treated with abacavir 160 mg/kg). There was no significant difference between the controls and abacavir treatment groups. Acetylcholine-induced endothelium-dependent relaxation in rat basilar arteries was unaffected when rats were treated with both dosages of abacavir (Fig 12A and 12B). Endothelium-independent relaxation was also studied using sodium nitroprusside. 10 nM of sodium nitroprusside induced a relaxation response of 64.4 ± 6.3% and 66.8 ± 8.5% in control arteries and arteries incubated with abacavir for 24 h, respectively. Sodium nitroprusside-induced relaxation was 48.9 ± 7.4% and 52.3 ± 8.2% in control rats and rats treated with 160 mg/kg of abacavir, respectively.

## Discussion

Unexpectedly and opposite to our hypothesis that abacavir may impair endothelial function, our results showed that abacavir induced endothelium-dependent relaxation of rat basilar arteries. These findings are novel, thus further experiments were carried out to investigate the mechanism of abacavir-induced relaxation. It is well established that the endothelium releases endothelium-dependent relaxing factors such as NO, PGI_2_ and EDHFs upon stimulation [29,30]. In the present study, different pharmacological agents were used to determine which factors were responsible for the effect of abacavir. Studies have demonstrated that NO is the major factor responsible for the basal tone of rat basilar arteries [31–33]. The present study consistently showed that the relaxation induced by abacavir could be attenuated only by the NO synthase inhibitor, L-NAME. This finding indicated that abacavir-induced relaxation was solely NO-mediated. PGI_2_ and EDHF were probably not involved in abacavir-induced relaxation as the presence of indomethacin and TRAM-34 and UCL 1684 did not reverse the relaxation response. These findings are unsurprising as basilar arteries are considered to be large cerebral arteries, and it has been demonstrated that the EDHF response is more prominent in smaller arterioles [32,34].

It is well known that NO released from endothelial cells can activate soluble guanylyl cyclase in vascular smooth muscle cells, which catalyzes the formation of cGMP. The increase in intracellular cGMP level in turn activates protein kinase G leading to the stimulation of a cascade of phosphophorylation/dephosphorylation of proteins which relaxes vascular smooth muscle cells [35–38]. Consistent with this phenomenon, our results showed that abacavir-induced relaxation was inhibited by the guanylyl cyclase inhibitor, ODQ, and the protein kinase G inhibitor, KT 5823. Soluble guanylyl cyclase is highly expressed in the cerebral vasculature [32,39–41]. In our experiments, an enzyme-linked immunosorbent assay was used to measure the effect of abacavir on intracellular levels of cGMP. Arteries were incubated with abacavir for 20 min as the arteries were fully relaxed by abacavir at around 20 min. We postulated that cGMP should reach a maximum level within 20 min. Our data showed that abacavir treatment increased the intracellular level of cGMP in basilar arteries, and that the increased level of cGMP was abolished by ODQ. Taken together, we are convinced that activation of the NO/soluble guanylyl cyclase/cGMP/protein kinase G signaling pathway is the major mechanism by which abacavir induces relaxation.

The cAMP signaling pathway is another common mechanism involved in the vasodilatory response. An increase in intracellular cAMP level can activate protein kinase A, which induces the relaxation of vascular smooth muscle cells. Interestingly, the cAMP signaling pathway may ‘crosstalk’ with the cGMP signaling pathway. This crosstalk mechanism is present in the regulation of cerebral vasodilatation [41]. Therefore, it is not impossible that cAMP may play a part in abacavir-induced relaxation. However, our results showed that the adenylyl cyclase inhibitor, SQ 22583, and the PKA inhibitor, KT 5720, did not alter abacavir-induced relaxation. In good agreement with these results, the results of immunoassay showed that abacavir did not increase the intracellular concentration of cAMP in rat basilar arteries. These data suggest that the cAMP/PKA signaling pathway does not contribute significantly to abacavir-induced relaxation.

The relaxation effect of abacavir occurred in <1 min. However, abacavir uptake by endothelial cells was very slow, and the amount of abacavir which accumulated within endothelial cells in 1 min was negligible. This finding indicated that the vasorelaxing effect of abacavir was probably mediated through membrane receptors rather than intracellular mechanisms. Abacavir is a purine nucleoside analog. Adenosine, an endogenous purine nucleoside, can dilate different vascular beds through stimulation of adenosine receptors [42,43]. It was suspected that abacavir may induce relaxation through stimulation of adenosine receptors. Interestingly, abacavir-induced relaxation was attenuated by CGS-15943 (a non-selective adenosine receptor antagonist) and ZM-241385 (a selective adenosine A_2_ receptor antagonist). This finding indicated that the abacavir effect was mediated by adenosine A_2_ receptors. Several studies have demonstrated that adenosine induces vasodilatation through a direct action on vascular smooth muscle cells. However, adenosine receptors are also found in endothelial cells, and adenosine can dilate blood vessels partly through endothelium-dependent mechanisms [44]. By acting on different A_2_ receptors on endothelial cells and vascular smooth muscle cells in coronary arteries, adenosine can induce endothelium-dependent NO-mediated relaxation and endothelium-independent ATP-sensitive potassium channel-mediated relaxation, respectively [45]. We demonstrated that abacavir-induced relaxation was endothelium-dependent, thus we can assume that abacavir stimulated A_2_ receptors on endothelial cells, which subsequently released NO causing relaxation of vascular smooth muscle. However, the adenosine receptors (A_2A_ or A_2B_) involved in the abacavir-induced response could not be confirmed. A_2A_ and A_2B_ receptors are predominantly expressed on endothelial cells, and A_2B_ receptors have been reported to mediate the adenosine-induced dilatation of cerebral arterioles [45–47]. Although ZM-241385 shows a higher affinity to A_2A_ receptors compared with A_1_ and A_3_ receptors, it is suspected that ZM-241385 can also bind to A_2B_ receptors [48,49]. In addition, it is accepted that activation of A_2_ receptors leads to an increase in intracellular cAMP [50,51]. However, the present study showed that abacavir increased the level of cGMP (but not cAMP) in basilar arteries. The reason for this discrepancy is not known, but may be due to differences in vascular beds. In accordance with the results in the present study, it has been reported that adenosine relaxes intracerebral arterioles through cGMP-dependent mechanisms rather than cAMP-dependent mechanisms [43].

It is not known if abacavir acts upon adenosine receptors directly or indirectly through the increased extracellular concentration of endogenous adenosine. The extracellular adenosine concentration is controlled by (i) ecto-5′ nucleotidase, which produces adenosine through conversion from adenosine monophosphate and (ii) an adenosine transport system which removes extracellular adenosine by taking it up into cells. The present study showed that abacavir had no effect on ecto-5′ nucleotidase. The major adenosine transport systems in endothelial cells are ENT1 and ENT2. The former can transport NRTIs such as didanosine and zalcitabine, whereas ENT2 can transport azidothymidine at low affinity [52,53]. The present study showed that abacavir (>100 μM) inhibited adenosine uptake by ENT1 and ENT2. Theoretically, the blockade of ENT1 and ENT2 should increase the local concentrations of adenosine in the vicinity of adenosine receptors. However, this proposed mechanism does not account for the relaxation effect of abacavir as 10 μM abacavir relaxed basilar arteries by >50%, however, this concentration of abacavir was not high enough to inhibit ENT1 and ENT2. Therefore, we postulate that abacavir may stimulate adenosine A2 receptors directly as they have a similar chemical structure to purine nucleosides.

Studies have shown that short-term (24 h) treatment of porcine arteries with antiviral drugs causes impairment of relaxation [9,54]. Therefore, the effect of short-term abacavir treatment on vascular contractility was investigated. An abacavir concentration of 10 μM was used in the present study as this is the plasma concentration of abacavir [9]. Short-term treatment of abacavir did not affect acetylcholine-mediated and sodium-nitroprusside-mediated relaxation of basilar arteries. Our results did not agree with those from a recent study which reported that endothelium-dependent vasorelaxation in porcine pulmonary arteries and human pulmonary arterial endothelial cells was impaired when the arteries were incubated with abacavir for 24 h [9]. Indeed, several clinical studies have demonstrated that antiviral therapy is linked to a higher incidence of pulmonary hypertension [55,56]. This discrepancy may be due to the use of different experimental models. We also suspect that abacavir-induced endothelial dysfunction may be specific in certain vascular beds (e.g., pulmonary arteries but not basilar arteries).

HIV-infected patients receive life-long antiviral therapy. Therefore, the long-term impact of abacavir on vascular health was also studied. Two dosages of abacavir, 16 mg/kg/day and 160 mg/kg/day, were used; 16 mg/kg is the daily dosage used clinically [57]. The dosage for humans and rats may not be identical due to differences in absorption, metabolism or excretion. However, pharmacokinetic data on abacavir in rats are not available, thus a higher dosage (ten-fold higher than the dosage for humans) was also used. Although abacavir therapy increases the risk of cardiovascular events such as myocardial infarction and stroke, the present study showed that the treatment of rats with abacavir (16 mg/kg/day and 160 mg/kg/day) for 28 days had no effect on the endothelium-dependent and endothelium-independent relaxation of basilar arteries. Indeed, our experimental results were consistent with a cohort study which showed that abacavir did not significantly increase the prevalence of cerebrovascular accidents [8].

The major objective of the present study was to determine the impact of abacavir on vascular contractility. We **showed that s**hort-term and long-term **treatment of abacavir did not inhibit endothelium-dependent and endothelium-independent relaxation. I**nterestingly, we found that abacavir may interact with adenosine A_2_ receptors on the endothelium, thereby inducing NO release which causes relaxation of blood vessels. Nitric oxide level was not measured in our study due to technical difficulties in isolating enough endothelial cells from rat basilar arteries for biochemical assay. HUVECs may be used instead of rat basilar endothelial cells, however, it is generally agreed that the capacity of HUVECs to produce NO is relatively low and a detectable amount of NO can only be measured after several hours of drug incubation. In theory, abacavir-induced NO release should potentiate the acetylcholine-mediated relaxation of blood vessels, however, this phenomenon was not observed in our study when the arteries were incubated with abacavir for 24 h or when the rats were treated with abacavir for 4 weeks. This may be due to two reasons. First, the adenosine receptors may be downregulated or desensitized after treatment with abacavir. Further study is required to prove this hypothesis. The second explanation is that abacavir was washed away before the isometric tension of arteries was measured in the myograph system (as mentioned in the methodology). The threshold concentration of abacavir which induced a direct vasorelaxing effect was ~10 μM, which is comparable to the plasma concentration in patients who receive the clinical dosage of abacavir [9]. The implications of this direct vasorelaxing effect of abacavir is not known, however, at the very least, the increase in cardiovascular risk caused by abacavir is unlikely to be due to endothelial dysfunction.

It has been reported that NRTIs cause endothelial dysfunction in rats and mice by increasing the concentration of the superoxide anion [58,59]. However, abacavir is a novel NRTI which does not inhibit polymerase γ in mitochondria. Theoretically, it is unlikely that abacavir causes mitochondrial toxicity, thus the production of reactive oxygen species is reduced [29,60–62]. This probably explains why endothelial dysfunction in the presence of abacavir was not observed.

An authoritative review has strongly recommended the inclusion of abacavir in the initial treatment regimen for HIV-infected patients [63]. In fact, the US Food and Drug Administration (FDA) is currently re-evaluating the overall risks and benefits of abacavir. This evaluation may result in the need to revise product labeling. As a precaution, the underlying risk of coronary heart disease or stroke should be considered when prescribing antiretroviral therapies (including abacavir), and action should be taken to minimize all modifiable risk factors (e.g., hypertension, hyperlipidemia, diabetes mellitus, smoking). Prevention of the adverse cardiovascular effects of abacavir is a long-term goal. The present study showed that abacavir relaxes, but does not constrict blood vessels, indicating that the cardiovascular risk caused by abacavir may not be related to its effect on vascular tone. Instead, increased platelet activity by abacavir may be one of the major mechanisms leading to higher cardiovascular events [64,65]. Theoretically, abacavir may stimulate endothelial cells to release NO, which should prevent platelet aggregation. However, the uptake of abacavir into platelets is high. Unlike that in vascular cells where the cellular uptake of abacavir is negligible, abacavir can be converted into the active metabolite carbovir triphosphate inside the cytoplasm of platelets. Carbovir triphosphate is able to blunt the increase in intraplatelet cGMP through the inhibition of guanylyl cyclase and thus acutely enhances platelet aggregation [65]. Nevertheless, the vasorelaxing effects of abacavir may still be a concern. The incidence of abacavir hypersensitivity is 4.3%, of which 7% of hypersensitivity cases demonstrate symptoms of hypotension [66]. It is not known whether the relaxation effect of abacavir may potentiate the severity of hypotension. In addition, there is evidence of the ability of adenosine A_2_ receptors to heteromerize with other G-protein coupled receptors, such as dopamine, glutamate, cannabinoid and ATP receptors in the striatum [67]. The roles of these G-protein coupled receptors in blood vessels are not yet understood. It is unclear whether the agonistic effect of abacavir on adenosine A2 receptors may modulate the functions of these receptors in blood vessels.
